# Tissue distribution of a plasmid DNA encoding Hsp65 gene is dependent on the dose administered through intramuscular delivery

**DOI:** 10.1186/1479-0556-4-1

**Published:** 2006-01-30

**Authors:** AAM Coelho-Castelo, AP Trombone, RS Rosada, RR Santos, VLD Bonato, A Sartori, CL Silva

**Affiliations:** 1 Departamento de Bioquímica e Imunologia, Faculdade de Medicina Universidade de São Paulo, Ribeirão Preto, SP, Brazil; 2 REDE-TB: Rede Brasileira de combate à tuberculose, USP, Riberiao Preto, São Paulo, Brasil; 3 Instituto de Biociências, UNESP, Botucatu, São Paulo, Brasil

## Abstract

In order to assess a new strategy of DNA vaccine for a more complete understanding of its action in immune response, it is important to determine the *in vivo *biodistribution fate and antigen expression. In previous studies, our group focused on the prophylactic and therapeutic use of a plasmid DNA encoding the *Mycobacterium leprae *65-kDa heat shock protein (Hsp65) and achieved an efficient immune response induction as well as protection against virulent *M. tuberculosis *challenge. In the present study, we examined *in vivo *tissue distribution of naked DNA-Hsp65 vaccine, the Hsp65 message, genome integration and methylation status of plasmid DNA. The DNA-Hsp65 was detectable in several tissue types, indicating that DNA-Hsp65 disseminates widely throughout the body. The biodistribution was dose-dependent. In contrast, RT-PCR detected the Hsp65 message for at least 15 days in muscle or liver tissue from immunized mice. We also analyzed the methylation status and integration of the injected plasmid DNA into the host cellular genome. The bacterial methylation pattern persisted for at least 6 months, indicating that the plasmid DNA-Hsp65 does not replicate in mammalian tissue, and Southern blot analysis showed that plasmid DNA was not integrated. These results have important implications for the use of DNA-Hsp65 vaccine in a clinical setting and open new perspectives for DNA vaccines and new considerations about the inoculation site and delivery system.

## Introduction

It had been discovered that plasmid DNA encoding a protein antigen could serve as an effective immunogen. Since then, DNA-based vaccines have garnered attention for their potential as alternative treatments for various diseases [1]. For vaccinologists, the main advantages of this approach are the adjuvant effects provided by unmethylated CpG motifs in the plasmid backbone and by CD8 T cell activation. However, despite the efficacy of naked DNA vaccines, different results concerning the biodistribution, the kind of cells involved in the uptake process, as well as the *in vivo *genome integration and the time of antigen expression have been demonstrated for each DNA construct and delivery method (2–8). The analysis of such factors could advance the understanding of this vaccination strategy and lead to methodological improvements, such as the use of lower amounts of plasmid without altering the immune response.

Our group has focused on intramuscular administration of naked plasmid DNA encoding the *Mycobacterium leprae *65-kDa heat shock protein (HSP65) and has demonstrated that this form of plasmid administration results in a good immune induction, as well as provides protection against virulent *M. tuberculosis *challenge [9]. The protection was attributed to the induction of a cellular immune response dominated by antigen-specific T lymphocytes that not only produced interferon-γ but also were cytotoxic to infected cells [10]. In addition, in heavily infected mice, vaccination with Hsp65-encoding DNA resulted in a pronounced therapeutic effect altering the relatively inefficient immune response which produces bacterial stasis, into an efficient response that was able to kill the bacteria [9]. This vaccine also showed good results in studies employing the prime-boost strategy against both experimental [11] and bovine tuberculosis [12].

As previously mentioned, the plasmid biodistribution and genome integration, as well as its *in vivo *persistence and antigen expression, have been little explored in these DNA vaccination models. Nevertheless, these aspects are very important for the design of new delivery strategies and biosafety.

Integration into the host cell genome could produce insertional mutagenesis, which would have the potential of activating or inactivating genes. In addition, the plasmids used in Hsp65 DNA vaccine have, in their nucleotide sequence, the SV40 virus origin of replication, which could permit the *in vivo *replication of the plasmid. These phenomena can be verified by distinguishing between prokaryotic methylation patterns present in plasmid DNA and the eukaryotic genome of the host, which can be considered another safety measure.

Some aspects of biodistribution have been analyzed with other delivery systems and doses, such as vector models or naked DNA encapsulated in a delivery vehicle [13-17]. The results have shown that a widespread biodistribution of the vector occurs in all systems. However, the time analyzed after immunization was variable and it is difficult to compare the results.

In the present study, we observed tissue distribution of naked plasmid DNA-Hsp65 vaccine and RNA expression by 6 months following intramuscular administration in mice. Additionally, we investigated whether the plasmid DNA replicates or integrates into the mammalian genome when residing in tissues over the long term.

## Materials and methods

### Plasmid DNA construction and purification

The construction of a pcDNA3 plasmid containing the cytomegalovirus (CMV) promoter and a cDNA encoding the HSP65 gene for *M. leprae *(pcDNA3-HSP65) has been previously described [9]. Plasmid DNA was purified as described in the EndoFree plasmid purification handbook (Qiagen, Ltd., Crawley, UK). Spectrophotometric analysis revealed the 260/280 nm ratios to be ≥ 1.80. The purity of DNA preparations was confirmed on a 1% agarose gel.

### Immunization

BALB/c mice (three animals for each time point) evaluated in the study were 6–8 weeks old and were obtained from the Animal Facilities of the University of Sao Paulo, School of Medicine at Ribeirão Preto. The mice were maintained under standard laboratory conditions. The naked plasmid DNA doses used were 4, 20 and 100 μg/mouse (w/v) in 25% PBS-sucrose (100 μl total volume) and were administered by intramuscular injection into the right quadriceps muscle at two separate sites in the same muscle. As negative control mice were immunized with control vector in PBS-sucrose (three mice per group).

### Isolation of DNA and RNA

At various time points following the administration of naked pcDNA3-Hsp65 or pcDNA3 DNA vector (data not shown), samples of several tissue types, including muscle, draining lymph node, spleen, lung, liver, kidney and thymus, as well as a single-cell suspension of bone marrow, were obtained. The samples were treated with Trizol reagent (Invitrogen, Carlsbad, CA, USA) and total RNA and DNA were isolated according to the manufacturer protocols. Subsequently, RNA was extracted with chloroform and precipitated with isopropyl alcohol. The DNA was isolated by ethanol precipitation of the interphase and phenol phase. The precipitated DNA was washed with 0.1 M sodium citrate followed by 75% ethanol. The total extracted RNA and DNA were dissolved in nuclease-free water (Invitrogen). Total DNA of *E. coli *was isolated by the same protocol.

### RT-PCR

Total cellular RNA (10 μg/ml) was reverse transcribed using oligo(dT) primers and reverse transcriptase (Invitrogen) according to the manufacturer instructions. The contaminating plasmid DNA was removed by treatment with DNAse I, amplification-grade (Invitrogen). The cDNA (2 μg) was amplified for 35 cycles at 94°C for 30 seconds, 60°C for 45 seconds and 72°C for 1.5 minutes, using the primer pairs 5'- ACC AAC GAT GGC GTG TCC AT-3' and 5'- TAG AAG GCA CAG TCG AGG-3', resulting in a 400-bp cDNA encoding Hsp65, or the primer pairs 5'- GTG GGC CGC TCT AGG CAC CAA-3'and 5'- CTC TTT GAT GTC ACG CAC GAT TTC-3', resulting in a 450-bp cDNA encoding β-actin. In order to avoid cross-contamination, all procedures, including the PCR, were performed in separate laminar flow hoods.

### Plasmid rescue procedure

Total DNA was extracted from all tissues using Trizol reagent according to the manufacturer instructions. From the total DNA isolated, 1 μg was transformed into competent *E. coli *DH 5α as previously described [3,5,19] and plated on Luria-Bertani agar plates using an ampicillin selection for the plasmids (100 μg/ml). Rescued plasmids were analyzed by restriction mapping (data not shown), insert release and by PCR using primers (5'- ATG GCC AAC ACA ATT GCG TAC-3' and 5'- TTG AGC AGG TCC TCG TCG TAC TCA C-3') that amplified a 1500-bp fragment, under the same conditions as for the PCR reaction describe above. The nucleotide sequencing of rescued plasmid was carried out with a DNA sequencing kit (Big Dye Terminator Cycle Sequencing Kit; Perkin-Elmer, Norwalk, CT, USA) and an ABI PRISM 3100 Genetic analyzer (Applied Biosystems, Foster City, CA, USA) according to manufacturer instructions. The primers used were T7 or BGH. Sequence homologies were obtained by using the Basic Local Alignment Search Tool (National Center for Biotechnology Information, Bethesda, MD, USA). Transformation of *E. coli *with 1 μg of wild type pcDNA3-hsp65 was used as positive control of transformation.

### Methylation status of plasmid DNA

Of the total cellular DNA obtained from muscle, 1 μg was digested for 4 hours with 10 units of Nde I and the dam methylation pattern was then analyzed [18] with Mbo I or Dpn I (Invitrogen) overnight at 37°C. Ten μl of the reaction mixture were used to perform a PCR for 35 cycles at 94°C for 30 seconds, 60°C for 45 seconds and 72°C for 1.5 minutes, using T7 and BGH primer pairs (5'-TAA TAC GAC TCA CTA TAG GG- 3' and 5'-TAG AAG GCA CAG TCG AGG- 3'). Amplified DNA was analyzed by ethidium bromide staining after 1% agarose gel electrophoresis. The methylation pattern of *E. coli *was used as a positive control.

### Southern blot

After one month of inoculation with naked plasmid pcDNA3-Hsp65, genomic DNA was isolated from the livers of immunized mice, as well as from those of nonimmunized mice, using Trizol reagent (Invitrogen). The samples were digested with Nde I overnight or were left undigested. From each, 10 μg of total cellular DNA were subjected to electrophoresis on a 0.8% agarose gel. The Southern blot analysis was carried out using Gene Images™ (Amersham Pharmacia Biotech, Uppsala, Sweden), and hybridization bands were revealed using naked pcDNA3 vector (1 μg/ml) labeled with the random prime labeling module (Amersham).

## Results

### Biodistribution of plasmid pcDNA3-Hsp65 and detection of message of Hsp65

**Figure 1:**
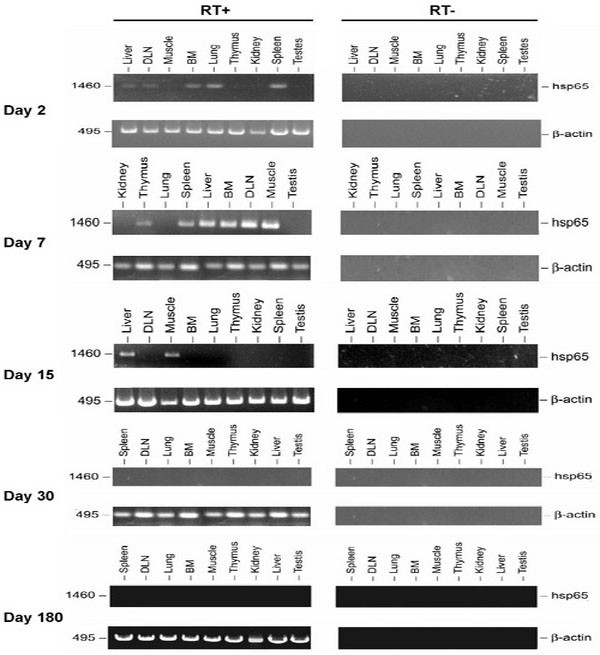
Tissue *distribution of Hsp65 message*. The presence of Hsp65 message in various tissue samples obtained from BALB/c mice after intramuscular immunization with 100 μg of pcDNA3-HSP65 in 25% sucrose. Total RNA (10 μg) isolated from each tissue was treated with DNase I and subjected to RT-PCR amplification with HSP65 or β-actin primers (RT+). As an RNA quality control, β-actin was amplified. No products (HSP65/β-actin) were seen when total RNA in the absence of reverse transcription was subjected to PCR amplification (RT-). All RT-PCR products were analyzed by agarose gel electrophoresis and visualized by ethidium bromide staining. The results were obtained from one mouse and are representative of three independent experiments. RT-PCR from the material obtained of mice (three animals for each point) immunized with the vector (pcDNA3) was negative in all analysis.

To identify plasmid DNA-Hsp65 and expression of the encoding protein at remote sites after intramuscular injection, we used RT-PCR and bacterial transformation approaches. Animals received intramuscular injections of naked pcDNA3-Hsp65 or the control vector (pcDNA3) and then were sacrificed at various time points. Multiple tissue samples were collected for detection of plasmid DNA and Hsp65 message. On day 2 after inoculation, RT-PCR tissue analysis demonstrated the presence of Hsp65 transcripts in nearly all the tissue samples examined, with the exception of kidney and lung (Fig. 1; Table 1). On day 7, the Hsp65 message was still present in liver, muscle, bone marrow, draining lymph node and spleen. However, by day 15, the Hsp65 message could be detected only in muscle and liver tissue (Fig. 1; Table 1). The Hsp65 transcripts were not detected in tissues from animals immunized with the plasmid DNA vector (data not shown). However, plasmid DNA-hsp65 was widespread when the injection was done with the higher dose but not with the lower doses (Table 2). Moreover, it was possible to rescue plasmid DNA from mice injected with the higher plasmid dose in all tissue analyzed until 6 months (table 1). Interestingly the number of plasmid rescues increased over 30 days following immunization in nearly all tissues (Table 2).

**Table 1:** Biodistribution of pcDNA3-Hsp65 and detection of hsp65 message *in vivo*.

	Days after intramuscular inoculation									
	
	RNAm (message)					Plasmid DNA				
	
Tissue types	2	7	15	30	180	2	7	15	30	180
Muscle	+	+	+	-	-	+	+	+	+	+
DLN	+	+	-	-	-	+	+	+	+	+
BM	+	+	-	-	-	+	+	+	+	+
Spleen	+	+	-	-	-	+	+	+	+	+
Liver	+	+	+	-	-	+	+	+	+	+
Lung	-	-	-	-	-	+	+	+	+	+
Kidney	-	-	-	-	-	+	+	+	+	+
Thymus	+	-	-	-	-	+	+	+	+	+

Detection of plasmid and hsp65 mRNA from tissues isolated at indicate time point following intramuscular inoculation of 100 μg pcDNA3hsp65. DNA and total RNA were isolated from the tissues using Trizol reagent per manufacturer's protocol. The presence of plasmid DNA was done by bacterial transformation and the hsp65 message by RT-PCR. DLN: draining lymph nodes; BM: Bone marrow

**Table 2:** Number of bacterial colonies obtained after transformation of tissue DNA after immunization with different doses.

pcDNA3-hsp65 doses	Time after immunization	Number of colonies/μg total DNA^a^							
		
		Muscle	DLN	BM	Liver	Spleen	Lung	Kidney	Thymus
100 μg	2	51	45	113.4	120.7	94.7	34.3	19.7	23.3
	7	45.6	92.7	96.7	102.7	110.7	13.7	14	16.7
	15	60.3	106.4	93	93.3	101.3	14	9.7	19.3
	30	64.7	98.4	109	98.7	101	6.3	1.3	12.7
	180	9.4	11.4	15.7	93.3	17.7	3	1	9.7

20 μg	2	2	3	1	0	3	0	0	0
	30	0	0	0	0	0	0	0	0
	180	0	0	0	0	0	0	0	0

^a ^This represents the total number of colonies obtained using 1 μg of total tissue DNA when the transformation efficiency was about 10^4 ^colonies/μg plasmid DNA (1 μg pcDNA3-Hsp65 wild type, data not shown). The total number of colonies is given as the median of values obtained in three independents transformation assay (three mice).

### Identification of the rescued plasmid

**Figure 2:**
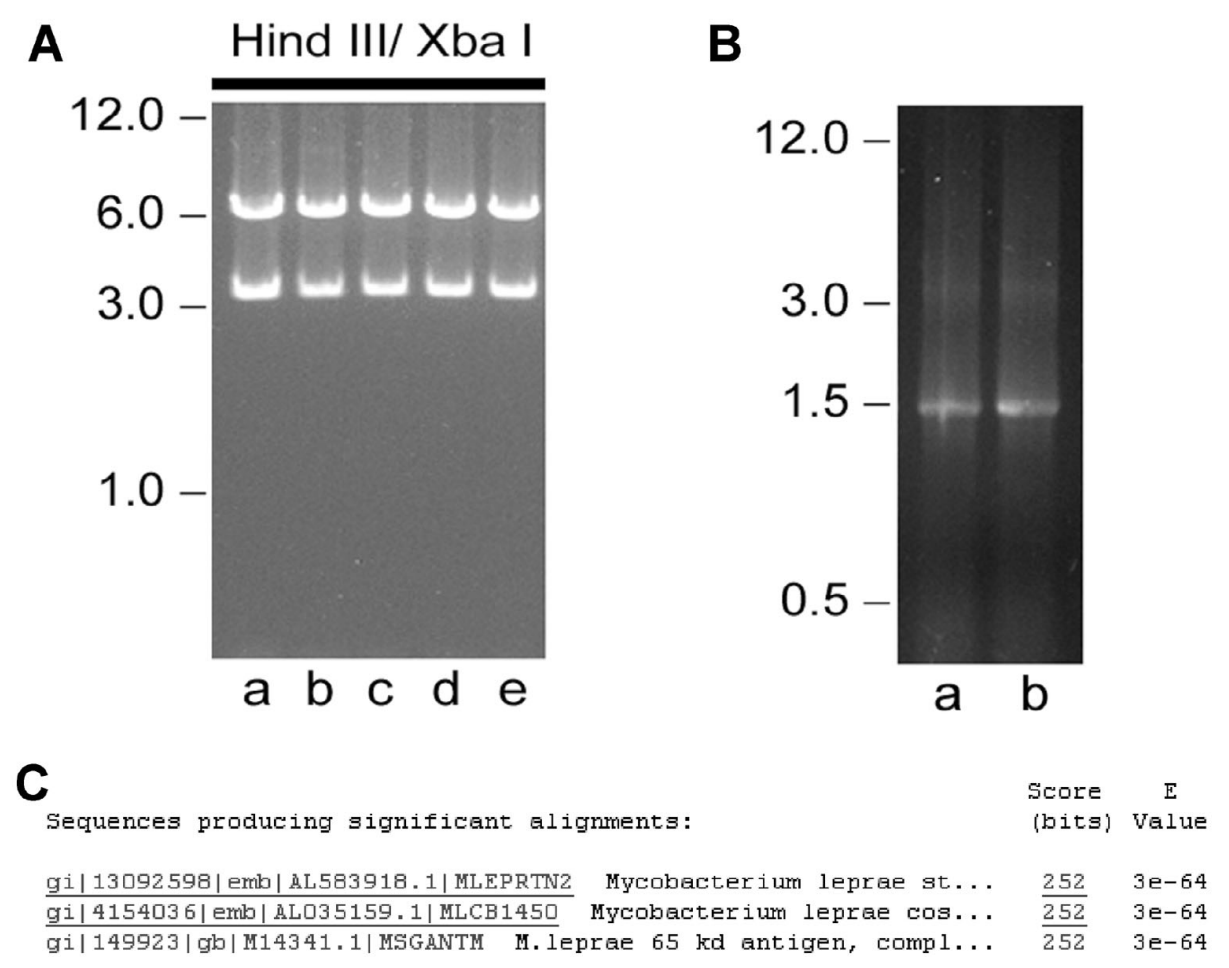
*Identification of plasmid DNA *rescued. Nature of plasmid DNA obtained after transformation of cellular DNA from tissues of mice 2 days after i.m. immunization with pcDNA3-Hsp65. *Escherichia coli *DH5-α was transformed with 1 μg of total DNA from tissues of mice previously immunized with pcDNA3-Hsp65. Plasmid DNA was recovered from ampicillin-resistant colonies. (A) Agarose gel showing plasmid DNA digested overnight with Hind III and Xba I: wild-type pcDNA3-Hsp65 (lane a); plasmid DNA recovered from muscle (lane b); plasmid DNA from bone marrow (lane c) plasmid DNA from liver (lane d); plasmid DNA from spleen (lane e). (B) PCR analysis of rescued plasmid using HSP65 primers: Wild type pcDNA3-Hsp65 (lane a); plasmid rescued from muscle of immunized mice (lane b). The mobility of DNA size standards (λ DNA cut with Hind III) are shown on the left. (C) Identification of nucleotide sequence of plasmid DNA rescued from muscle. Sequence analyses were performed using the blastn program from BLAST.

In order to clarify that the rescued plasmids, after bacteria transformation, were pcDNA3-Hsp65, we digested the plasmids from different tissues (muscle, bone marrow, liver and spleen), or wild type plasmid (as a positive control) using restriction enzymes that release the insert. The digestion pattern indicated that ampicilin-resistant colonies obtained from these tissues, contained the pcDNA3-Hsp65 plasmid (Fig. 2A). The identity of pcDNA3-HSP65 was also confirmed by PCR, using Hsp65-specific primers and plasmid DNA sequence analysis (Figure 2B and 2C). These results confirmed that the plasmids rescued in our analyses were pcDNA3-Hsp65 demonstrating the specificity of the methodology.

### Methylation status of plasmid DNA in muscle

**Figure 3:**
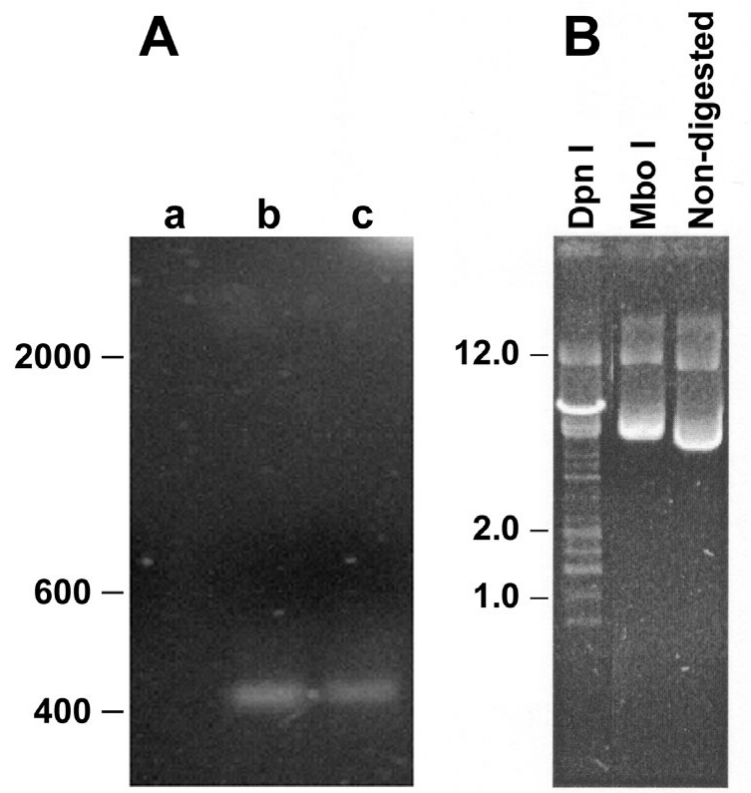
*Persistence of DNA adenine methylase site methylations (dam) of pcDNA3-HSP65 in muscle at 6 months after immunization*. (A) Approximately 1 μg cellular DNA obtained from muscle of immunized mouse were digested with Nde I and Dpn I (lane a), Nde I and Mbo I (lane b), or with Nde I alone (lane c) and amplified by PCR using Hsp65 primers. The samples were submitted to electrophoresis on a 1% agarose gel (B) The positive control was done using *E.coli *DNA digested with Dpn I, Mbo I or non-digested to show the *dam *methylation pattern. The mobility of DNA size standards (l DNA cut with Hind III) are shown on the left.

In order to determine whether pcDNA3-Hsp65 replicated in tissue over the long-term, muscle DNA preparations were digested with Nde I and then with Dpn I (Fig. 3A, lane a) or Mbo I (Fig. 3A, lane b) prior to PCR analysis. All DNA samples were digested with Nde I because PCR amplification with linear plasmid yielded more product than circular plasmid DNA that was undigested (data not shown). Samples of muscle DNA obtained six months after inoculation were subjected to the above procedure. Amplified fragments appeared only in those samples digested with Nde I alone (Fig. 3A, lane c) or with Mbo I alone (Fig. 3A, lane b). No amplified fragments were evident in samples digested with Dpn I prior to PCR (Fig. 3A, lane a). We obtained similar results using total cellular DNA from liver and spleen samples (data not shown). The positive control of *dam *methylation in *Escherichia coli *is indicated in the Figure 3B.

### Genomic integration

**Figure 4:**
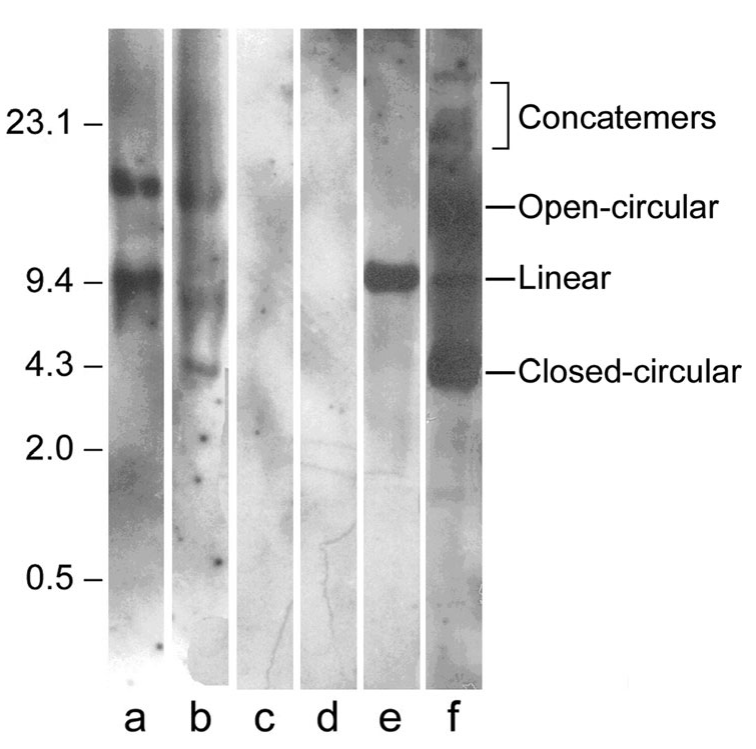
*Analysis of the pcDNA3-Hsp65 genome integration*. Samples of liver tissue from mice immunized with 100 μg of pcDNA3-HSP65 (lanes a and b) and from nonimmunized mice (lanes c and d) (negative control) were submitted to Southern blot after Nde I digestion. Lanes e and f correspond to wild-type plasmid digested with Nde I or undigested, respectively. The bands were detected using pcDNA3 labeled with chemiluminescent reagent. The multiple forms of plasmid DNA are indicated in the figure. The samples were loaded in a same gel and the lanes not used were removed.

To exclude the possibility that pcDNA3-Hsp65 was integrated into the host cell genome, we performed Southern blot analyses in liver tissue samples from immunized and nonimmunized mice. The pcDNA3-Hsp65 from the liver tissue samples was digested with Nde I to become a linear plasmid, which was evidenced by a single 9000-kb band (Fig 4, lane e). The undigested plasmid (Fig. 4, lane f) presented the characteristic bands corresponding to multimeric forms of nonintegrated plasmid DNA. This same band pattern was observed in the genomic DNA of immunized mice (Fig. 4, lanes a and b), showing that pcDNA3-Hsp65 was not integrated. The liver tissue from nonimmunized animals presented no bands (Fig. 4, lanes c and d).

## Discussion

It has been proposed that the long-term expression of a foreign gene is one of the principal indicators of gene therapy success. From the standpoint of vaccines, however, short-term expression may prevent the systemic tolerance induced by repeated exposure to antigen [20]. There are a variety of factors that can potentially affect plasmid gene expression, including antigen-specific immune response and cytokine-regulated promoter function [reviewed [21]]. Using only one dose of 100 μg pcDNA3-Hsp65 by intramuscular delivery, we observed the presence of Hsp65 message in several tissue samples until 7 days after injection, including secondary lymphoid organs. After this period the Hsp65 message was detected only in muscle and liver (Figure 1). Since our assay was not quantitative, we did not determine the amount of Hsp65 message in each tissue; but its presence in each material for a limited time was shown. However we believe that the success of our vaccine against experimental tuberculosis could be due to another two doses of plasmid at 15 days. This schedule of immunization could preserve the Hsp65 message at lymphoid organs resulting in the induction of specific and efficient immune response [9].

Even though the Hsp65 message was not detected after fifteen days, the plasmid DNA could still be found in all analyzed tissues including lung, DLN, spleen, liver, bone marrow, kidney, muscle and thymus for at least six months post-inoculation. These findings indicate that plasmid DNA-Hsp65 disseminated widely throughout the body and persisted as a plasmid DNA form or produced lower doses of message not detectable in our RT-PCR assay (Table 1 and 2). After two days of immunization, about 100 ampicillin resistant colonies/μg genomic DNA could be obtained from different tissues. However, the number of the ampicillin-resistant colonies decreased at later time points, suggesting that the levels of plasmid DNA was also reduced (Table 2). At six months a lower frequency of recovered plasmid DNA was observed in kidney, lung and thymus (Table 2). The presence of plasmid DNA in the thymus could be a concern, since the expression of antigen in this tissue could induce tolerance by deletion of Hsp65-specific T cells altering the induction of Hsp65 immune response after intramuscular immunization schedule. However, the message was detected only two days after immunization in almost all tissues analyzed and the number of plasmids rescued decreased after six months, suggesting that the plasmids could have been damaged or digested by endonucleases. Interestingly, the presence of plasmid DNA was observed in different tissues for longer time points, even in the presence of lower numbers of colonies. These results are important because they show that even after widespread biodistribution of plasmid, the detection was reduced after 6 months. Furthermore, there are substantial data provided by our laboratory demonstrating that the presence of naked DNA in different organs does not change the histological pattern, suggesting the absence of inflammatory response in these tissues (manuscript in preparation, Deison Soares personal communication).

To assure that the plasmid rescued from different tissues was pcDNA3-Hsp65, we analyzed several plasmids by three different methods: restriction pattern (data not shown) and insert released, PCR and nucleotide sequence. Figure 2 illustrates the results of the same samples obtained from different tissues. These experiments were done to determine the identity of the plasmid DNA and to avoid false-positive results. The results confirmed that the plasmid rescued was the pcDNA3-Hsp65.

The widespread distribution of plasmid DNA throughout the body, regardless of the method and route of administration, has been previously reported [4,6,8,15,16,22]. Currently, the mechanism involved in this widespread biodistribution of plasmid DNA is not completely defined. It has been speculated that this may occur as a result of the transport of free plasmid DNA as well as its transport by transfected cells [22]. Nevertheless, the widespread biodistribution cannot be restricted to a particular mechanism or cell type, since after immunization with naked DNA it can be taken up by different cells like CD11b+ (2), CD11c (23,24), CD11c and CD19 (3), reaching distant sites. However, we cannot exclude the transport of plasmid DNA by serum or lymph.

Based on our results we suggest that the widespread biodistribution can also be correlated with the plasmid dose injected. To evaluate this possibility, some mice were immunized with lower doses of plasmid DNA-Hsp65 (4 and 20 μg/mouse). The results presented in Table 2 showed that in mice receiving the 20 μg dose, the plasmid remained in some tissues such as muscle, lymph nodes and bone marrow and was recovered only after 48 h after inoculation, whereas in mice that received the lower dose of 4 μg, the plasmid DNA was not recovered from any of the tissues analyzed (data not shown).

The limited biodistribution in mice that received lower doses cannot be attributed to the sensitivity of the method used, since by using plasmid DNA labeled with fluorescent dye (10–12 μg/mouse) we showed a similar pattern of biodistribution [3]. Consequently, we suggest that the widespread biodistribution is also dose correlated. These results are important for the development of new vectors, enabling the use of lower doses and thereby reducing the risks in the clinical application of DNA vaccines. Nowadays, one of the alternatives to reduce the amount of the plasmid administered is the delivery of it by micropheres [14,16,17] or liposomes [7]. However, the plasmid DNA is not easily released from these complexes and this can arrest the induction of an efficient immune response. Therefore, new approaches, such as that described in [25] can be an alternative to reduce the plasmid DNA dose without altering the immune response.

Interestingly, the Hsp65 message was consistently detected in muscle and liver tissue samples at day 15. In addition, the number of colonies in the liver was quite reduced (Table 2) which could, in part, explain the detection of Hsp65 message in such tissue fifteen days after DNA injection. The long-term persistence of plasmids in liver and muscle tissues has also been observed by other authors using different plasmid constructs [5]. However, other authors obtained different results after using alternative routes of plasmid administration, such as the intranasal [26]. As suggested by Wolff *et al*. (1992), the long-term antigen expression by muscle cells can be related to structural features, such as multinucleated cells. It is possible that these tissue characteristics are responsible for the prolonged presence of the message to Hsp65 in the muscle tissue. However, there is no satisfactory explanation for the Hsp65 long-term message expression in liver tissue yet, but we must be clear that the backbone of plasmid DNA can also take part in message expression.

The recovery of plasmid DNA from eukaryotic cells using bacterial transformation is a simple, fast and sensitive method, which was suitable for our objective of verifying the presence or absence of plasmid DNA in different tissues independent of the copy number in the tissues. On the other hand we also detected the plasmid in some tissues by PCR indicating that both methods showed the same results (data not shown).

In general, plasmid DNA used in genetic vaccines possesses an SV40 sequence of replication and could replicate *in vivo*. Replicative synthesis of plasmid DNA in mammalian cells can be evidenced by the appearance of molecules lacking the DNA adenine methylase-dependent (DAM) adenosine methylation [[27]. Plasmid DNA from bacteria contains a methylated adenosine within the GATC recognition sites for Dpn I and Mbo I. The Dpn I cleaves the site more efficiently if the adenine is methylated, whereas Mbo I cleaves the site more efficiently if the adenine is unmethylated [18,27]. If the plasmid DNA replicates in mammalian cells, then the bacterial methylation pattern is lost. The pattern of methylation in *E.coli *was shown in Figure 3B, as a positive control. Prokaryotic DNA was cleaved by DpnI as expected, but not by Mbo I (Fig. 3B), to assure the *dam *methylation pattern. The methylation of plasmid rescued from muscle is shown in Figure 3A. This result revealed that the bacterial methylation pattern of the injected pcDNA3-Hsp65 DNA was unchanged after having resided in muscle for at least six months, indicating that this plasmid DNA did not replicate *in vivo*. These results are in agreement with those from previous studies using different plasmid DNA vaccines [5,28]. In addition, the lack of plasmid replication *in vivo *provides a higher degree of safety for gene therapy vaccination.

The recovery of ampicillin-resistant colonies from the tissues of pcDNA3-Hsp65-immunized mice after the transformation of total cellular DNA suggests that some extrachromosomal plasmid DNA was maintained for at least 6 months. Southern blot analysis was done with liver and muscle (data not shown) samples at 30 days after immunization due the higher number of plasmid rescued by bacterial transformation in liver sample. Furthermore, the number of plasmid rescued at 180 days was comparable to the number of plasmids obtained at 30 days. The results displayed in figure 4 showed that the plasmid did not integrate into the BALB/c genome. In general, plasmid integration into genomic DNA occurs by tandem repeats [30], and when released from the genome the plasmid shows a linear form in Southern blot. On the other hand, when the plasmid DNA is not integrated it has the blueprint of undigested DNA. The pcDNA3-Hsp65 showed a pattern similar to undigested plasmid DNA when compared to wild type plasmid DNA used in immunization (Figure 4, lane a and f). These results indicated that pcDNA3-Hsp65 was not integrated into the mouse genome at the time point analyzed.

The frequency of integration into the cellular genome could be affected by several factors, such as the plasmid sequence, the presence of *chi*-like elements [29], *Alu *segments [30] and minisatellite regions [31]. However, the integration of bacterial plasmid DNA is not quite so simplistic. The mammalian genome appears to possess a mechanism to protect its integrity [32]. In addition, the results provided by Ledwith *et al. *(2000), using different plasmid constructs suggest that the risk of integration of plasmid DNA vaccines following intramuscular inoculation is negligible. Therefore, the use of plasmid DNA in gene therapy can be safer than vector systems. At the present time, the mechanisms involved in the non-integration of pcDNA3-Hsp65 could not be definitively characterized. However, our results suggest that this vaccine is safe for clinical use and indicate that the use of a plasmid containing the Hsp65 gene is reliable for gene therapy purposes as well as for vaccination in a clinical setting. In addition, the results of long time biodistribution/dose after intramuscular delivery were observed and described for the first time herein. Accordingly, we consider that our findings open not only new perspectives for DNA vaccines but also lead to new considerations about the inoculation site and delivery systems.
